# Neuroplasticity and Mechanisms of Action of Acute and Chronic Treatment with Antidepressants in Preclinical Studies

**DOI:** 10.3390/biomedicines12122744

**Published:** 2024-11-29

**Authors:** Gilberto Uriel Rosas-Sánchez, León Jesús Germán-Ponciano, Gabriel Guillen-Ruiz, Jonathan Cueto-Escobedo, Ana Karen Limón-Vázquez, Juan Francisco Rodríguez-Landa, César Soria-Fregozo

**Affiliations:** 1 Centro Universitario de Los Lagos, Universidad de Guadalajara, Lagos de Moreno 47460, Jalisco, Mexico; giluriel.30@gmail.com; 2 Laboratorio de Neurofarmacología, Instituto de Neuroetología, Universidad Veracruzana, Xalapa 91190, Veracruz, Mexico; lgerman@uv.mx (L.J.G.-P.); analimon@uv.mx (A.K.L.-V.);; 3 Programa Investigadoras e Investigadores por México-CONAHCYT-Instituto de Neuroetología, Universidad Veracruzana, Xalapa 91190, Veracruz, Mexico; gguillen@uv.mx; 4 Instituto de Ciencias de la Salud, Universidad Veracruzana, Xalapa 91190, Veracruz, Mexico

**Keywords:** neuroplasticity, antidepressants, acute and chronic treatment, depression, mechanisms of action, neurotrophic factors

## Abstract

Pharmacotherapy for depression includes drugs such as monoamine oxidase inhibitors (MAOIs), tricyclic antidepressants (TCAs), selective serotonin reuptake inhibitors (SSRIs), noradrenaline (NA) and serotonin (5-HT) reuptake inhibitors (NaSSAs), and atypical antidepressants; these drugs exert differentially beneficial effects on symptoms of depression after acute and chronic treatment in animal models. Said effects are established through neuroplastic mechanisms involving changes in neurogenesis and synaptogenesis as result of the activation of intracellular signaling pathways associated with neurochemical and behavioral changes. Antidepressants increase the synaptic availability of monoamines (monoaminergic hypothesis) such as 5-HT, NA, and gamma-aminobutyric acid (GABA) by inhibiting their reuptake or degradation and activating intracellular signaling pathways such as the responsive element binding protein (cAMP-CREB) cascade, which regulates the expression of genes related to neuroplasticity and neurogenesis, such as brain-derived neurotrophic factor (BDNF), in various brain structures implicated in depression. The aim of this review is to analyze the mechanisms of action of different antidepressants and to compare the effects of acute and chronic treatment on neuroplasticity in animal models of depression. A thorough search was conducted in PubMed, Scopus, and Web of Science, focusing on studies since 1996 with keywords like antidepressants, acute and chronic treatment, neuroplasticity, and experimental depression. Studies included had to investigate antidepressant effects experimentally, with full-text access, while excluding those that did not. Data extraction focused on study design, findings, and relevance to understanding treatment differences. Only high-quality, peer-reviewed studies were considered to ensure a comprehensive synthesis of current knowledge.

## 1. Introduction

The therapeutic effect of antidepressants involves complex mechanisms that go beyond the traditional hypothesis of monoaminergic deficits to include changes in neuroplasticity. Neuroplasticity is the adaptive ability of the brain to reorganize and form new connections under normal and pathological conditions [1]. Depression is associated with dendritic neuronal atrophy and a reduction in glial cells and dendritic arborization in neurons of the prefrontal cortex and hippocampus, among other brain structures. In addition, an increase in dendritic branching of neurons of the cerebral amygdala was observed [2]. The neurotrophic hypothesis states that treatment with antidepressants promotes neuroadaptive changes in the brain that counteract some symptoms of depression [3]. Chronic antidepressant treatment promotes complex mechanisms at the cellular, molecular, and structural levels of neurons via intracellular signaling pathways involved in survival and neuroplasticity. Acute administration of fast-acting antidepressants such as ketamine and psilocybin can activate various signaling pathways that modulate glutamate release; this mechanism is regulated by GABAergic neurons and astrocytes, which activate the transcription of BNDF and Homer1a in response to synaptic plasticity. Chronic antidepressant treatment stimulates the function of cAMP-CREB, a transcription factor that regulates the expression of genes involved in neuroplasticity, cell survival, and cognition [1], for example, cAMP-CREB, mitogen-activated protein kinase (MAPK), *BFos*, and serum response factor (SRF) [4].

This promotes neurogenesis, dendritic branching, and synaptogenesis in the hippocampus and prefrontal cortex and reverses the pathological effects of stress and depression [5]. The cAMP-MAPK-CREB cascade of BDNF is involved in dendritic remodeling, neurogenesis, and cell survival [6]. Chronic stress has been reported to decrease neuroprotective factors such as BDNF, which negatively affects neuroplasticity and increases neuronal atrophy. This phenomenon leads to a reduction in synaptic contacts and brain volume [2,7]. In contrast, other subcortical regions, such as the amygdala, show hypertrophy in male rats exposed to the despair model with defeat and stress, possibly contributing to the anxiety and altered emotions characteristic of this disorder [8]. These findings emphasize the importance of understanding the neurobiological mechanisms underlying depression in order to develop more effective and targeted therapeutic approaches.

Treatment alternatives for depression have been tested in preclinical studies with the aim of improving efficacy and safety. This research is crucial, considering that the response rate to existing treatments is only 50%. This response rate refers to the first treatment with antidepressants, usually SSRIs. However, efficacy and side effects are thought to influence patients’ decision to continue treatment [9]. Antidepressants such as agomelatine, citalopram, escitalopram, fluoxetine, sertraline, and vortioxetine were more tolerable than other antidepressants, with acceptance rates reflected in odds ratios (ORs) ranging from 0.43 to 0.77. On the other hand, drugs such as amitriptyline, clomipramine, duloxetine, fluvoxamine, reboxetine, trazodone, and venlafaxine had the highest discontinuation rates, with ORs ranging from 1.30 to 2.32. This study included a total of 116,477 patients, 522 from randomized controlled clinical trials (RCTs) conducted between 1979 and 2016, comparing 21 antidepressants or placebo [10]. In this sense, the effect of chronic treatment with antidepressants has been confirmed in animal models, such as forced swimming, where the effect of relatively low doses of fluoxetine on immobility was observed at a dose of 1 mg/kg fluoxetine in male Wistar rats during 21 and 28 days of treatment. This dose increases the latency to first immobility and decreases the time of immobility, which is indicative of an increase in the animal’s motivation and a decrease in behavioral despair [11,12]. In addition, an increase in BDNF has been observed specifically in the hippocampus and prefrontal cortex of mice [13] and an increase in the expression of 5-HT_1A_ and 5-HT_2A_ receptors in the raphe, hippocampus, and cerebral cortex of the rat [14]. This increase in expression of the receptors is related to an increase in the availability of 5-HT, indicating the establishment of the therapeutic effect. However, acute treatment, especially at high doses administered 24, 5, and 1 h before the forced swim test, has been shown to alter behavioral indicators of hopelessness in rats. These effects appear to be related to an increase in the levels of neurotransmitters such as GABA, dopamine (DA), NA, and 5-HT without necessarily causing plastic changes in the brain structures that control emotional processing [15,16]. However, clinical and preclinical studies have shown that antidepressants have a long latency period for the onset of therapeutic effect, together with the multiple side effects associated with their use over long periods of time [17]. These findings on antidepressants drugs have led to the exploration of new pharmacological strategies targeting mechanisms other than those specified in the monoaminergic hypothesis of depression.

The aim of this review is to provide a comprehensive understanding of the mechanisms of action of different antidepressants and to determine the differences between acute and chronic treatment with antidepressants on neuroplasticity in animal models of depression. To prepare this review, a comprehensive bibliographic search of PubMed, Scopus, and Web of Science was conducted using specific keywords, such as antidepressants, acute treatment, chronic treatment, forced swimming, neuroplasticity, and experimental depression. The search was refined to include only studies published since 1996. Inclusion criteria focused on research articles, reviews, abstracts, and position statements that investigated the effects of different types of antidepressants drugs in preclinical studies during chronic and acute treatment, as well as their mechanism of action and changes in neuroplasticity. Exclusion criteria excluded those studies that did not provide access to the full text.

Data extraction followed a structured approach, capturing critical information on study design, key findings, and the relevance of each study to understanding the differences in antidepressant effects between acute and chronic administration and the potential mechanism of action of each effect. We assessed the quality and validity of the studies based on methodological rigor, reproducibility, and peer-reviewed status. This approach ensured that only high-quality studies were considered and enabled a comprehensive synthesis of the current state of knowledge.

## 2. Overview of Depression Disorder

According to the World Health Organization, depression is a mental disorder characterized by a depressed mood or a lack of interest in activities for prolonged periods of time [18]. This debilitating emotional state is accompanied by symptoms such as cognitive impairment, hopelessness, feelings of emptiness, changes in sleeping and eating patterns, difficulty concentrating, and suicidal thoughts [19]. The effects of depression extend beyond the person affected and significantly affect their family, social, and community relationships [18]. Depression is one of the leading causes of disability worldwide, affecting approximately 3.8% of the world’s population, or around 280 million people [20,21,22,23]. In patients with other diseases, depression exacerbates the disease burden and has a negative impact on quality of life [24,25]. The recent COVID-19 pandemic, which began in 2020, has further exacerbated this situation, with factors such as the birth of a child and the loss of a loved one contributing to increased rates of depression [26,27,28].

Neurobiological studies have shown a close link between depression and changes in neuroplasticity, particularly in neuronal atrophy and the reduction in dendritic branches in important brain regions such as the prefrontal cortex and the hippocampus. A model has been proposed in which prolonged stress decreases neuroprotective factors such as BDNF, negatively affects neuronal plasticity, and accelerates neuronal atrophy. This process leads to a reduction in synaptic contacts and brain volume [2,29]. On the other hand, other subcortical areas, such as the amygdala, show hypertrophy associated with depression, which may contribute to the anxiety and altered-emotions characteristics of this disorder [9]. These findings emphasize the importance of understanding the neurobiological mechanisms underlying depression to develop more specific and effective therapeutic approaches.

Depression has a multifactorial origin; recent studies show that it is the result of a complex interaction between biological, psychological, and social factors. In this sense, depression occurs in different subtypes, each with different characteristics and requiring specific treatments [30]. Among the most important subtypes is major depression, for which the most common pharmacological treatment is the use of SSRIs. However, a significant percentage of patients do not achieve remission with this treatment [31]. Within this group, seasonal affective disorder, which is characterized by recurrent symptoms such as sadness and lack of energy during certain seasons, especially autumn and winter, responds well to treatment with fluoxetine and bupropion, as well as phototherapy and vitamin D [32,33]. In contrast, melancholic depression, which is characterized by a marked loss of pleasure, a lack of reactivity to positive stimuli, and a worsening in the morning, has been shown to respond better to tricyclic antidepressants than to SSRIs [34]. In atypical depression, which is associated with increased emotional reactivity, increased appetite or weight, hypersleep, and sensitivity to rejection, a better response to MAOIs has been observed, although SSRIs are also effective [35,36]. Psychotic depression, characterized by hallucinations, intense guilt, and severe psychomotor disturbances, generally requires a combination of antidepressants and antipsychotics [37]. In addition, the subtype of dysthymia, also known as persistent depressive disorder, is defined as a mild and persistent form of depression that interferes with daily life and must persist for at least two years for a diagnosis. In these cases, SSRIs and SNRIs have been shown to be effective [38]. This classification of subtypes reflects not only the pathophysiological heterogeneity of depression but also the different response of each subtype to different classes of antidepressants.

Despite this complexity and the numerous biological factors involved, antidepressants can have adverse effects that reduce adherence to treatment [39,40]. Low adherence and side effects have driven the search for therapeutic alternatives [41,42,43]. In this context, preclinical research on treatment alternatives aims to improve the efficacy and safety of antidepressant pharmacotherapies. As response rates to current treatments are only around 50% [9], research into alternatives, such as herbal medicines, are crucial for improving treatment outcomes. Given the chronic nature of depression and the need for both acute and long-term treatment solutions, it is crucial to continue exploring these alternatives to address the significant global burden of depression.

## 3. Pharmacological Treatment of Depression

Early studies in major depressive disorder (MDD) patients showed decreased levels of the monoamines 5-HT, DA, and NA in the brain, leading to the so-called “monoaminergic hypothesis”, which led to the development of several classes of antidepressants mainly targeting the monoaminergic system [44,45]. The monoaminergic hypothesis for depression, based in part on the antidepressant efficacy of serotonergic drugs, has not found sufficient support in the studies conducted to date [46]. Therefore, there is an urgent need to find new non-serotonergic agents with greater pharmacological efficacy and fewer side effects. MAOIs were the first agents to show antidepressant activity by increasing the availability of monoamines in the synaptic cleft by preventing their degradation by monoamine oxidase (MAO) enzymes [47]. However, the use of MAOIs was restricted due to adverse effects and their reported toxicity [48,49].

Pharmacological treatment of MDD is based on the use of serotonergic drugs despite their limited efficacy. Several mechanistically novel drugs have been developed in recent years, but many of them fail in clinical trials. Several hypotheses have been proposed to explain the pathophysiology of MDD, suggesting that physiological processes such as neuroplasticity, circadian rhythms, and metabolism are potential targets [47].

Despite their limited efficacy, these antidepressants, especially the SSRIs, are still the most important drugs for the pharmacological treatment of MDD. Approximately 35–50% of patients do not respond to treatment [50,51]. In addition, lethargy, sedation, and sexual dysfunction are side effects of serotonergic antidepressants, making it difficult for patients to adhere to the required treatment regimens [52,53]. The time lag of at least two weeks to improvement of depressive symptoms when using currently available medications and the lack of response in treatment-resistant depressed patients are also issues that need to be addressed [54].

Another class of drugs are the TCAs and tetracyclic antidepressants. TCAs such as imipramine cause an improvement in depressive state in some patients by inhibiting presynaptic NE and 5-HT reuptake transporters, leading to an increase in the concentration of NE and 5-HT in the synaptic cleft [47]. However, cyclic antidepressants can also act on other postsynaptic receptors (α-adrenergic, histaminergic, and cholinergic), which has been associated with adverse effects such as dizziness and memory impairment [49,55].

In the search for antidepressants with improved efficacy and effectiveness, pharmaceutical companies began researching ligands that selectively inhibit 5-HT reuptake. This led to the development of fluoxetine, the first SSRI to be approved by the Food and Drug Administration (FDA) [56]. Since then, other SSRIs have been shown to be effective and are prescribed as first-line treatment for depression. SSRIs work by increasing the availability of 5-HT in the synaptic cleft, specifically by inhibiting 5-HT reuptake transporters. SSRIs have fewer side effects than TCAs, which typically include sexual dysfunction, insomnia, and loss of appetite [45,49].

Another class of medications used to treat MDD are atypical antidepressants, which differ in their mechanism of action from other treatments and often act on multiple targets. 5-HT and NA reuptake inhibitors (SNRIs), such as venlafaxine and duloxetine, have been developed to selectively inhibit both the 5-HT and NA transporters [55]. There is evidence that SNRIs may be more effective than SSRIs in the treatment of MDD. Unfortunately, this effect appears to be specifically related to venlafaxine, which, however, has a higher discontinuation rate due to side effects [57,58,59].

Atypical or multimodal antidepressants act on other neurotransmitter systems. Bupropion, for example, is a DA and NA reuptake inhibitor that has a higher affinity for the DA reuptake transporter [60], while agomelatine is a melatonin receptor agonist and an inhibitor of certain serotonergic receptors [61]. Another class of antidepressants is referred to as “noradrenergic and serotonergic specific antidepressants” or NaSSAs. These drugs, represented primarily by mirtazapine, antagonize the a2-adrenergic receptor and inhibit certain serotonergic receptors [62]. Vortioxetine, one of the drugs recently approved by the FDA, inhibits 5-HT reuptake by inhibiting its transporter and has a high affinity for several types of 5-HT receptors [63]. The common side effects of atypical antidepressants are usually mild and include nausea, dry mouth, insomnia, nervousness, and low libido.

Recently, some antidepressants have been launched, such as es-ketamine (Spravato^®^) [64], a glutamatergic antagonist, the combination of dextromthorphan, an N-methyl-D-aspartate (NMDA) receptor antagonist, with bupropion (Auvelity^®^) [65], and brexanolone (Zulresso^®^), a positive allosteric modulator of GABA_A_ [66]. Although these are new drugs for the treatment of MDD, they were developed for specific indications, making it difficult to use them for specific types of depression. For example, Zulresso^®^ is only prescribed for postpartum depression; Spravato is only indicated for patients who do not respond to drug treatment for depression and who may experience sedation and cognitive impairment as side effects; and Auvelity^®^ has addiction potential, as dextromethorphan is known to have this problem. These new drugs can be seen as a significant advance in the pharmacotherapy of MDD. However, they have limitations due to side effects, so the search for antidepressants with better efficacy and efficiency for the treatment of MDD should continue [47]. In addition, clinical, biological, and environmental factors are crucial to understand the heterogeneity in response to antidepressant treatment. Clinical characteristics such as severity of illness, comorbidities, and family history as well as social characteristics such as age, gender, and education of patients are crucial to predict variability in treatment response; moreover, patient-related factors such as forgetfulness, comorbidities, and misconceptions about the disease and medication; medication-related factors, polypharmacy, side effects, pill burden and cost; healthcare-system-related factors, including physician–patient interactions, sociocultural factors such religious and cultural beliefs and stigma, and logistic factors were found to be the major factors associated with antidepressant non-adherence [67]. The most used antidepressants for the treatment of depression are listed in Table 1.

## 4. Antidepressants and Neuroplasticity

The main mechanism of action of antidepressants targets the synaptic availability of monoamines by inhibiting reuptake or degradation [43]. It is known that acute treatment modulates the expression of synaptic receptors. In addition, chronic administration of antidepressants leads to changes in the sensitivity of presynaptic and postsynaptic receptors [77]. The pharmacological response to chronic treatments in the clinic suggests that neuroplasticity mechanisms are necessary for the establishment of the therapeutic effects [78]. These mechanisms usually focus on the restructuring of neuronal synapses, a process known as neuroplasticity of the neurotransmission systems involved, mainly 5-HT, NA, and GABA. This process takes about 3 to 4 weeks [14], which explains the long latency period required to observe the therapeutic effects of antidepressant drugs.

The mechanism of action of drugs with antidepressant effects, such as TCAs, MAOIs, and SSRIs, have in common the enhancement of serotonergic neurotransmission [35], but their therapeutic effects are based on complex mechanisms [79]. Some of the mechanisms activate the adenylate cyclase–protein kinase A cascade and inhibit phospholipase C mechanisms that regulate gene expression. They also alter the expression levels of mRNAs of various receptors, neurotrophic factors, and neuropeptides [79]. Crystallographic studies of G protein-coupled receptors and neurotransmitter transporters have enabled us to understand the mechanism of antidepressants and to develop new drugs [80].

The molecular and cellular mechanisms by which antidepressants exert their effects are complex, i.e., they go beyond increasing the availability of monoamines in the synaptic space [81]. Acute administration of antidepressants can modulate the neuronal extracellular signal-regulated kinase/mitogen-activated protein kinase (ERK/MAPK) signaling pathway in the prefrontal cortex, which contributes to the restoration of neurogenesis and neuroplasticity [82]. Some antidepressants drugs such as fluoxetine and ketamine can bind to the trkB receptor and induce plastic changes in the hippocampus, prefrontal cortical regions, nucleus accumbens, and lateral habenula [83]. Chronic treatment, in turn, leads to greater activation of the cAMP system, which increases the expression of transcription factors, such as CREB, which regulate target genes of proteins such as BDNF [84]. In addition, it has been reported to increase neurogenesis in the adult hippocampus [83]. These changes in neuroplasticity strengthen synaptic connectivity and remodeling of neuronal circuits in the emotional pathways [81].

Several studies have reported that SSRIs ingestion in intact adult rodents induces long-lasting behavioral changes and neuroplasticity in the hippocampus and cortex [85,86]. In this sense, young rats (9 weeks old) treated with 10 mg/kg fluoxetine twice daily for three weeks showed greater motivation to explore novel environments in the Y-maze, with no effects on anhedonia and anxiety, as assessed by the sucrose test and the Elevated Plus Maze, respectively. However, an increase in the number of 5-bromodeoxyuridine-positive (BrdU+) cells, the density of dendritic spines in layer II/III pyramidal neurons of the medial prefrontal cortex, and BDNF/TrkB expression levels were observed. These changes can persist for up to 20 days after the last fluoxetine dose [86].

Chronic treatment with fluoxetine has been shown to induce neuroplasticity in various brain regions of rodents. In the somatosensory cortex of rats, it increases c-fos expression and dendritic spine density [87]. In mice, administration of 10 mg/kg fluoxetine over a two-week period led to a reduction in hopelessness, anhedonia, and anxiety. In addition, fluoxetine treatment decreased the deterioration of hippocampal neurons, and the number of dendritic spines tended to increase, which is attributed to synaptic plasticity [88]. It was also shown in the chronic social isolation model in rats that the administration of 5 mg/kg fluoxetine over a period of three weeks was able to reverse the behavioral effects induced by stress. On the other hand, the synaptosomal polysialic acid-neuronal cell adhesion molecule (plasticity-related molecule in the hippocampus) (PSA-NCAM), a molecular marker of plasticity, increases in the hippocampus of chronically isolated rats, an effect that is reversed by fluoxetine treatment [89]. However, the effects of fluoxetine in young rats differ from those in adults. While PSA-NCAM expression increases in various brain regions, such as the amygdala, the number of proliferating cells decreases in the subventricular zone. These and previous findings highlight PSA-NCAM as an interesting molecule for psychiatric research and a potential therapeutic target. [90].

These results show that the effect of fluoxetine on neuroplasticity varies according to treatment duration, age, and brain region, as the mechanism by which fluoxetine achieves the antidepressant effect is complex. Another study investigated the effect of chronic treatment with fluoxetine on neurogenesis and the expression of growth-associated protein 43 (*GAP43*), a synaptic protein, in the hippocampus of rats exposed to chronic unpredictable mild stress (CUMS), a behavioral model of depression. The results of this study showed that treatment decreased immobility in the forced-swim test and increased the expression of BrdU-positive cells and *GAP-43* (a protein associated with neuronal plasticity). However, fluoxetine has a greater effect on neurite outgrowth than on neurogenesis [91].

Other antidepressants such as agomelatine, venlafaxine, duloxetine, sertraline, and desvenlafaxine increase BDNF levels in the hippocampus and frontal cortex; BDNF is a regulator of neuroplasticity, and in this sense, BDNF signaling may induce changes and modulate the serotonergic system. Furthermore, it has been suggested that variations in the *Val66Met* gene may be associated with the therapeutic effect of antidepressants in MDD [92].

New treatments for depression based in some neurosteroids, such as allopregnanolone (brexenolone), produces some beneficial effects in the treatment of MDD symptoms, as they strongly modulate the function of GABA_A_ receptors, whose composition is altered in affective disorders. In addition, brexanolone induces neurosteroidogenesis through TSPO (18 kDa translocation protein) ligands, which may offer new treatment options due to the link between neurosteroidogenesis and synaptic plasticity [93].

On the other hand, ketamine increases the expression of molecules that modulate neuroplasticity, including changes in glutamatergic neurotransmission, as well as in AMPA receptors and in proteins and intramolecular signaling complexes such as target of rapamycin (mTOR), BDNF/TrkB. Unlike conventional antidepressants drugs, which take 4 to 6 weeks to establish their therapeutic effects, ketamine can improve mood within an hour of administration, and the effects last for up to 2 weeks. In this sense, two categories of mechanisms have been identified that contribute to the antidepressant effect of ketamine: molecular plasticity, which occurs within minutes/hours, and structural plasticity, which is observed over the days of treatment [94].

The behavioral models most used in preclinical research to study the mechanisms of action of diverse drugs or molecules with antidepressant potential include the forced swim test, sucrose preference test, and tail suspension, etc. Forced swimming, for example, is one of the most used and validated behavioral models for the study of drugs and substances with potential antidepressant effects, in which the hopelessness of the behavior is assessed. In this model, drugs or substances with antidepressant potential reduce immobility behavior and prolong the latency to the first immobility period, which is interpreted as an increase in the animal’s motivation to escape the stressful situation represented by the forced swim [11]. Furthermore, antidepressants that are ineffective when administered acutely have been shown to have an antidepressant effect when administered chronically to experimental animals [95]. This chronic administration is necessary if the effects of stress on immobility and changes in neuroplasticity are to be reversed [88,95]. The molecular mechanisms of neuroplasticity in the context of depression and antidepressant therapy are still unclear. However, it is known that antidepressants can promote neuroplasticity by altering cell signaling. This effect is mediated by an increase in the levels of neurotrophic factors such as BDNF, which activates tyrosine kinase receptors and triggers an intracellular cascade involving cAMP-dependent protein kinase A, MAPK, and other chemicals. These molecules activate transcription factors such as the cAMP-CREB and promote protein synthesis. The result is structural changes in certain regions of the brain. Table 2 describes the effects of antidepressants on neuroplasticity in animal models.

## 5. Behavioral Effects and Neuroplasticity in Acute Antidepressant Treatment: Preclinical Studies

In preclinical studies, it is possible to observe the pharmacological effects of high doses of antidepressants drugs during acute administration, a finding that is not possible to replicate in the clinic. There is evidence that acute treatment with antidepressants can have rapid effects on a subset of symptoms [105]. However, in humans, chronic administrations of antidepressant drugs during two to four weeks are necessary to achieve a desired pharmacological effect measured with the Hamilton scale [106].

Several studies have shown that a dose of 20 mg/kg of fluoxetine administered 23.5, 5, and 1 h before the FST reduced immobility behavior in male rats in the forced-swimming test through effects on the 5-HT_2C_ receptor [107]. On the other hand, [108] showed that fluoxetine at doses of 5 and 10 mg/kg reduced immobility and increased swimming behavior in ovariectomized female rats during treatment for 23.5, 5, and 1 h before the forced-swim test. Similarly, [16] reported that a dose of 10 mg/kg fluoxetine reduced immobility in female and male rats. However, low doses of 5 mg/kg fluoxetine do not have the same effect in male rats under the same dosing regimen [109]. Furthermore, antidepressants drugs that are ineffective when administered acutely produce antidepressant-like effects after chronic administration [95,109,110]. Such chronic administration in experimental studies is usually necessary to reverse the effects of stress on immobility [95,110].

Conventional antidepressants drugs represent the best pharmacological options; nevertheless, a high percentage of patients do not achieve sustained remission due to side effects or the long time it takes for the therapeutic effects to kick in. It is therefore necessary to explore more effective and safer drugs with a faster onset of action. In this sense, the fact that conventional antidepressants in preclinical research can produce different effects at effective or high acute doses, ranging from anxiogenic effects [111] to antidepressant effects in preclinical models after 1 to 3 administrations within 24 h, such as fluoxetine [14,15] and citalopram [111], in both sexes and different age groups [16]. These drugs have changed the paradigm of antidepressants, in which a long latency to therapeutic effect was considered an indispensable requirement, related to the establishment of slow-onset plastic changes in neuronal circuits regulating motivated behavior and associated with changes in neurotrophins levels [112,113], which will be described later.

On the other hand, recent studies in both preclinical and clinical research have identified new agents that can exert antidepressant-like effects after a single administration and over a period of several hours [114,115,116]. These agents are substances already known for other pharmacological properties, such as ketamine, which is used as an anesthetic and has been associated with antidepressant properties at the clinical level [117]; hallucinogens such as psilocybin and neurosteroids such as allopregnanolone also have been effective in the treatment of depression over a period of 24 to 36 h [118].

In preclinical studies, SSRI treatment has been reported to increase synaptic 5-HT availability to a limited extent when administered acutely (2 to 4 administrations). This is due to inhibitory effects on release mediated by somatodendritic 5-HT_1A_ receptors and terminal 5-HT_1B_ autoreceptors [111], although this inhibitory effect on release decreases after chronic treatment or due to antagonism of autoreceptors [119]. It has been known for more than two decades that acute antidepressant treatment with 10 mg/kg fluoxetine induces changes in the expression of the c-fos gene that differ from the expression patterns of the same gene after chronic treatments, helping to explain the plastic changes seen with chronic treatment [120]. For example, acute intraperitoneal administration of 10 mg/kg imipramine can reverse immobility time in the forced-swim test, with no observed effects on neurotrophin levels (NGF and BDNF) in the hippocampus and cerebral cortex [121]. Desipramine, reboxetine, bupropion, and pagyline reduce immobility in the TST model in intact mice in the same way as the SSRIs sertraline, fluoxetine, and paroxetine, but NA- and epinephrine-deficient mice by disruption of the DO-beta-hydroxylase gene did not show these changes, in which only citalopram reduced immobility [122], emphasizing the importance of the noradrenergic system for the acute effects of the first group of drugs. While a single administration of citalopram (30 mg/kg) showed no effect, three administrations of 10 mg/kg intraperitoneally (i.p.) within 24 h increased CREB phosphorylation in the hippocampus and decreased immobility time in the tail suspension test in mice, but not in CREBαΔ mutants, indicating its importance for the establishment of antidepressant effects. Moreover, these three administrations succeeded in blocking the hypothermia response induced by the serotonergic agonist 8-OHDPAT, suggesting that within 24 h, the 5-HT_1A_ and possibly 5-HT_7_ autoreceptors were desensitized [111]. However, these effects were not observed when the animals were exposed to another depression model, such as forced swimming with a single dose of citalopram (10 mg/kg, i.p.); in contrast, selective 5-HT_4_ receptor agonists, RS 67333 (1.5 mg/kg, i.p.) and prucalopride (2.5 mg/kg, i.p.), induce changes in CREB phosphorylation after only 3 days of treatment [123], emphasizing the potential of 5-HT_4_ receptor agonists as potentially fast-acting antidepressants [124].

On the other hand, it has been reported that chronic stress increases the release of glutamate and that the overactivation of its receptors is related to the development of depressive symptoms, so that one possible pathway of action of fast-acting antidepressants is the modulation of the glutamatergic system [125]. In this regard, ketamine is an NMDA receptor antagonist, and its antidepressant effect has been shown to be associated with modulation of AMPA and adenosine A1 receptors. This leads to an increase in synaptic plasticity and the formation of new synapses [126]. Ketamine is a non-competitive NMDA receptor antagonist that is commonly used as an anesthetic but also shows antidepressant effects when administered acutely. Acute administration of ketamine has shown antidepressant effects in rat [127] and mouse models [128,129,130]. For example, in the FST, high doses of imipramine (20 and 30 mg/kg) and ketamine (10 and 15 mg/kg weight) reduce immobility without altering motor activity and increase energy metabolism by increasing creatine kinase activity in the striatum, cerebral cortex, prefrontal cortex, and cerebellum [131]; but only the highest dose of ketamine increases BDNF and mTOR protein in the rat hippocampus [132]. The increase in BDNF was also observed in the prefrontal cortex of 14-month-old rats [133]. In addition, ketamine can reduce the levels of interleukins IL-6 and IL-1β and increase the 5-HT/tryptophan ratio in the hippocampus [134]. In mice, acute administration of ketamine also increased the expression of the neuropeptide pituitary adenylate cyclase-activating polypeptide (PACAP) in the dentate gyrus of the hippocampus, while blocking the signaling of this peptide attenuated the acute effects of ketamine in the tail suspension test, forced swimming, and sucrose consumption [135]. However, the antidepressant effects of ketamine are not always observed and depend on the dose, route of administration [128], and treatment protocol [136].

Ketamine promotes a cascade of cellular events that begin with the release of glu-tamate, the activation of AMPA receptors that activate mTOR-mediated plasticity, BDNF, and the synthesis of synaptic proteins that facilitate plasticity in structures such as the prefrontal cortex [137,138] or increase the activity of immature neurons without increasing neurogenesis [139]. Another example is psilocybin, in male Wistar–Kyoto rats, which exhibit a depression-like phenotype, a single administration of psychedelic substances, such as psilocybin (1 mg/kg) and LSD (0.15 mg/kg), resulted in antidepressant-like effects in the FST that appear to last longer than the effects of ketamine [140]. Although both psilocybin and ketamine induce changes in the dopaminergic, serotonergic, and GABAergic neurotransmission systems [141], a single administration of psilocybin leads to antidepressant-like effects in the FST that are related to changes in the neuroplasticity of the hippocampus and prefrontal cortex in mice [142]. These plastic changes appear to be related to the higher affinity of these psychedelics to the TrkB receptors for BDNF [143]. It should not be overlooked that psilocin and psilocybin have no effect in the forced-swim test in other cases, perhaps due to factors such as the genetic characteristics of the animals or the time after treatment at which the swim tests were performed [144,145].

**Figure 1:**
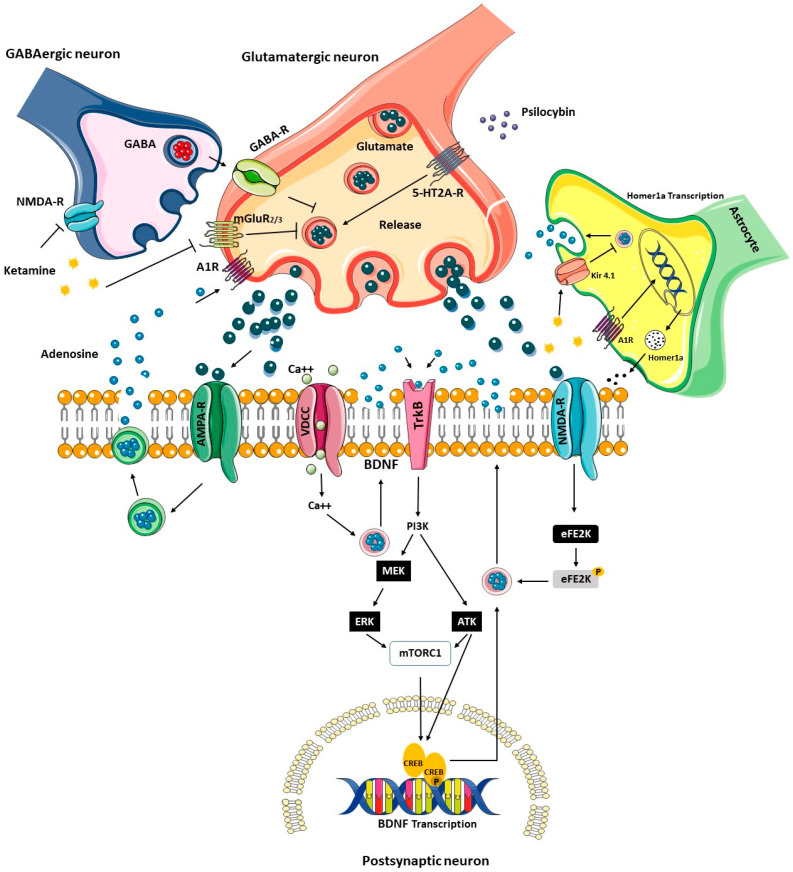
Signaling pathways activated by rapid antidepressants. Acute administration of ketamine and psychotropic drugs can activate various signaling pathways that modulate glutamate release. This mechanism is regulated by GABAergic neurons and astrocytes, which activate the transcription of BNDF and Homer1a in response to synaptic plasticity.

In addition, psilocybin can activate 5HT_2A_ receptors that promote the release of glutamate, a phenomenon that contributes to a rapid antidepressant effect by regulating various intracellular signaling pathways [126]. In this sense, it has been suggested that fast-acting antidepressants such as ketamine may activate the AMPA receptor and thus the release of adenosine, which in turn may activate the presynaptic adenosine A1 receptor and inhibit the release of glutamate. In addition, ketamine can disinhibit and block the NMDA receptor of GABAergic interneurons, a mechanism that decreases the release of GABA. This mechanism reduces the inhibition of glutamatergic neurons and causes the release of glutamate, which activates the postsynaptic AMPA receptors and promotes the entry of Ca2+ into the postsynaptic neurons. The entry of Ca2+ promotes the re-release of BDNF, which acts on the TrkB receptor, a phenomenon that promotes the activation of the PI3K-AKT and MEK-ERK pathways. Activation of mTORC1 promotes the transcription of BDNF. In contrast, one mechanism that promotes synaptic plasticity is the Ca2+-activated Calcium–calmodulin (CaM)-dependent protein kinase II (CAMKII) pathway, which promotes the transcription of *kcnq2* through the activation of the CAM-CAN-Akap5 complex. In addition, activation of this pathway promotes the transcription of BDNF via the CREB pathway. In addition, ketamine blocks the eukaryotic elongation factor 2-kinase-mediated inhibition of BDNF synthesis by inhibiting NMDA-type postsynaptic receptors. On the other hand, the effect of ketamine on glutamatergic neurons promotes the release of glutamate and thus improves synaptic transmission by inhibiting the mGlu2/3 receptor. In addition, in astrocytes, ketamine can inhibit the Kir4.1 channel and activate the adenosine A1 receptor, triggering the transcription and release of Homer1a, a phenomenon that contributes to the improvement of synaptic plasticity [126]. Figure 1 describes the mechanisms involved in the action of fast-acting antidepressants.

In 2019, the FDA approved brexanolone in an intravenous dosage form, whose active ingredient is the neurosteroid, allopregnanolone, for the treatment of postpartum depression. The decline in progesterone and neurosteroid levels in the postpartum period, which is seen as a kind of withdrawal of the effects of these substances, is part of the etiology of postpartum depression [146], an idea that had previously been proposed for the depression observed in premenstrual syndrome [147]. The antidepressant effect of allopregnanolone and other neurosteroids [148] has already been documented in preclinical studies in the FST in mice [149] and in male [150] and female rats without ovarian hormones [151] from the first acute administration by modulating the activity of the GABA_A_ receptor. Apparently, the mechanism of brexanolone involves activation of the GABA_A_ receptor at synaptic and extrasynaptic levels, leading to inhibition of circuits necessary for the treatment of postpartum depression [152]. The BDNF signaling pathway is also involved in these mechanisms, but they appear to be independent of AMPA receptor activation [153]. The mechanisms of this work and their similarity to other active agents of plant origin point to the possibility of new substances such as flavonoids, among which chrysin stands out [154]. The discovery of substances with mechanisms of action that produce virtually immediate antidepressant effects has led to the search for new substances such as reelin [155,156] with rapid action profiles that act by mimicking the mechanism of action of ketamine [115] or by novel mechanisms of action [157].

## 6. Behavioral Effects and Neuroplasticity in Chronic Antidepressant Treatment: Preclinical Studies

Depression is one of the most common mental disorders, characterized by persistent sadness and lack of interest or pleasure in previously rewarding or enjoyable activities [158]. According to the WHO, depression affects 3.8% of the world’s population, including 5.0% of adults and 5.7% of adults over 60 years. Approximately 280 million people worldwide suffer from depression [159]. SSRIs are widely used for the treatment and management of various mood disorders, particularly depression [160]. Three SSRIs, including fluoxetine, sertraline, and escitalopram, produce modest improvement (approximately 5–10%) in standardized depression scores in adolescent patients with moderate–severe depression without significantly increasing the risk of suicidal ideation or behavior [161].

It appears that SSRIs in chronic post-traumatic stress disorder (PTSD) reduce basal levels of daily cortisol and cortisol reactivity to stress, leading to an improvement in mood [162]. As we know, the main mechanism of action of SSRIs is the blockade of the 5-HT transporter (SERT), which causes an increase in the 5-HT in the synaptic cleft [163]. However, the therapeutic effects of SSRIs cannot be fully summarized in a simple inhibition of SERT [164]. Several studies have shown that SSRIs can alter BDNF levels. For example, long-term treatment with fluoxetine increases BDNF expression in the hippocampus of depressed rats [90] and in the olfactory bulb of depressed mice [165]. Sertraline and escitalopram also increase BDNF levels, which improves depression [166]. On the other hand, clinical studies have also shown this effect.

This increase in the formation of new neurons is crucial for the recovery of cognitive and emotional functions in patients with MDD [167]. Antidepressants also act by altering synaptogenesis, i.e., the formation of new synapses between neurons, and have effects on neuronal growth factors such as BDNF, which play an important role in promoting neuroplasticity and neuronal survival and reversing the reduction in volume of the hippocampus, a key structure of the limbic system in MDD [167]. The effects of drugs with antidepressant activity are described in Table 3.

Other studies have shown that chronic treatment with fluoxetine increases synaptic plasticity through effects on BDNF in the hippocampus and cortex. In addition, fluoxetine increases the volume of the hippocampus in people with MDD [168]. Similarly, antidepressants have been shown to increase levels of vascular endothelial growth factor (*VEGF*). This factor is crucial for promoting neurogenesis, angiogenesis, and neuronal survival as it promotes the formation of new neurons and synaptic connectivity [168]. For example, *VEGF* has been reported to regulate the effects of antidepressants such as lamotrigine, suggesting that this factor may serve as a biomarker to monitor response to antidepressant treatment [169]. On the other hand, other studies have shown that insulin-like growth factor (*IGF-1*) plays an important role in the effect of antidepressants. In patients with MDD, it has been observed that serum levels of this factor are elevated, while they decrease again after the onset of the effect of SSRIs. IGF-1 has neuroprotective properties in the CNS and promotes neurogenesis. *IGF-1* influences the effect of antidepressants because it activates the MAP/ERK and PI3K signaling pathways, which are crucial for neuronal survival and synaptic plasticity [170].

**Figure 2:**
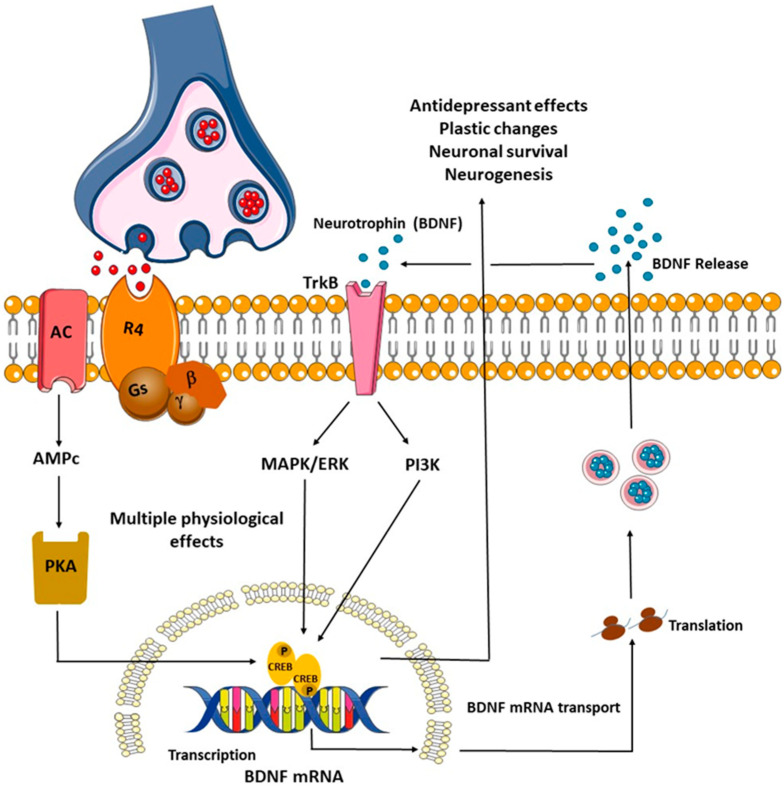
Signaling pathways that are regulated by chronic treatment with typical antidepressants such as the selective 5-hydroxytryptamine (5-HT) reuptake inhibitors (SSRIs). These block the reuptake of monoamines by the 5-HT transporter (SERT). This leads to the regulation of postsynaptic G-protein-coupled receptors coupled to a variety of second messenger systems, including the cAMP response element protein kinase A (PKA)-cAMP binding pathway (CREB). In addition, neurotrophic factor (BDNF)/tropomyosin-related kinase receptor type B (BDNF/TrkB) signaling promotes neuronal survival, plasticity, neurogenesis, and neuronal growth through activation of the MAPK/ERK (mitogen-activated protein kinases) PI3K (inositol 1,4,5-trisphosphate signaling pathways.

The relationship between BDNF and its receptor TrkB and antidepressants is an important area of research in the field of neuropharmacology. In this sense, a wide range of antidepressants such as SSRIs, dual antidepressants, and naturally occurring products such as chrysin (7,8-dihydroxyflavone) have been reported to increase BDNF levels by favoring the binding of this neurotrophins to its receptor TrkB, thereby activating several intracellular signaling pathways such as MAPK/ERK and PI3K, which are essential for promoting plastic changes in the brain and maintaining neuronal survival under stress conditions [171]. Similarly, conventional antidepressants, which generally focus on the monoaminergic system, may alter the function of NMDA receptors that are important for long-term potentiation, which is essential for the regulation of processes such as memory and for changes in emotional and affective state. Ketamine, an NMDA receptor antagonist, has shown a rapid and long-lasting antidepressant effect, while riluzole, an inhibitor of glutamate release that also increases the expression of the glutamate transporter in the glia, contributes to the elimination of depressive symptoms [172]. Figure 2 describes the signaling pathways that are regulated by chronic treatment with typical antidepressants.

**Table 3:** Effect of chronic treatment with antidepressants.

Drug	Dosage (Route of Administration, Dose and Duration of Treatment)	Experimental Subject	Experimental Model	Identified Effect	Mechanism	Reference
Ketamine Letrozole	Ketamine 5 mg/kg (i.p.). Letrozole 1 mg/kg. 7 days of treatment.	Adult female and male rats of the Sprague–Dawley strain.	FST	Reduction in immobility in FST in male with ketamine and reduction in FST immobility in female with letrozole. The combination of ketamine and letrozole reduces FST immobility in men.	It is assumed that they act via NMDA receptors and could modulate neurotrophic factors in the prefrontal cortex.	[173]
Levomilnacipran	30 mg/kg (i.p.). 14 days of treatment.	Male Wistar rats.	Depression induced with LPS 0.5 mg/kg for 2 weeks. SPT. FST. OFT.	Reduces immobility behavior in the FST. It reverses the increase in transcript levels of the proinflammatory cytokines IL-1β, INF-γ and TNF-α.	Inhibits the activation of the TLR4/NF-κB and Ras/p38 signaling pathways and modulates the ERK/CREB/BDNF pathway.	[174]
Fluoxetine	15 mg/kg/day (i.p.). 6 weeks.	Male Wistar Rats	Chronic social isolation (CSIS) (6 weeks) SPT FST	Increased sucrose consumption. Reduction in immobility time.	Expression of (CaMKK1). Phosphorylation CREB. Expression of BDNF.	[175]
Escitalopram Ibuprofen	Escitalopram 10 mg/kg (i.p.). Ibuprofen 40 mg/kg (i.p.). Combination of both. 21 days of treatment.	Adult male Sprague–Dawley rats.	Stress from restriction FST.	Reduction in immobility in FST with individual and combined treatment. Reduction in corticosterone levels. Increase in BDNF and p11 levels.	Positive regulation of BDNF and p11	[176]
Meloxicam Caffeic acid Sertraline	Meloxicam 3 mg/kg–1 mg/kg (i.p.). Caffeic acid 30 mg/kg–10 mg/kg (i.p.). Sertraline 5mg/kg (i.p.). Meloxicam 1 mg/kg + Caffeic acid 10 mg/kg (i.p.). 21 days of treatment	Adult male Sprague–Dawley rats	CUMS 6 weeks OFT. FST.	All treatments reduced immobility in FST. Caffeic acid inhibits NA reduction and increases Trp and MHGP. Meloxicam inhibits NA reduction and increases Trp, MHGP and Tyr.	Inhibition of COX-2 and reduction of 5-HIAA.	[177]
Bryostatin-1 Imipramine	Bryostatin-1 (20 µg/m^2^) Intravenous administration by tail Imipramine 15 mg/kg (i.p.). 5.5 weeks of treatment	Male Wistar Rats	Open-space swimming test Morris water maze Visible platform test.	Bryostatin-1 reduces immobility after 2 weeks of treatment. Bryostatin-1 restored the rats’ ability in spatial learning and spatial memory recall.	Protein kinase C (PKC)ε activation	[178]

## 7. Pharmacological Alternatives in the Treatment of Depression

One of the limitations of pharmacological treatment of depression is its side effects. Therefore, the search for alternatives through other pharmacological approaches, such as natural products and modulation of the inflammatory process and the microbiota, is promising [179]. In this sense, the presence of saponins, terpenes, and flavonoids in various herbal remedies has been shown to have antidepressant effects through the inhibition of SERT, NA, and DA [180]. On the other hand, it has been reported that plant molecules can regulate mood due to their antioxidant capacity. In this sense, rutin (quercetin-3-rutinoside) administered for 14 days at a dose of 80 mg/kg to rats with reserpine-induced depression was reported to prolong swimming time compared to the control group [181]. This effect is due to the antioxidant capacity of rutin and the decrease in acetylcholinesterase activity.

The combination of plant extracts with antidepressant activity in rats has shown a better effect when they were combined than when they were administered individually. In this regard, the combination of *Bupleurum chinese* DC (Chaihu) and *Paeonia lactiflora* Pall (Baishao) administered at a dose of 7.5 mg/kg for 28 days to rats with CUMS showed antidepressant effect by shortening the time of immobility and increasing the sucrose preference. The metabolomics results suggest that the antidepressant effect of the combination of Chaihui and Baishao is due to its ability to activate multiple signaling pathways and metabolites. The MAPK and arachidonic acid signaling pathways are critical for neuroplasticity, as they can influence synaptogenesis, inflammation, and growth factor expression, essential mechanisms for reducing the characteristic symptoms of depression [182].

Another natural product that has shown antidepressant properties in rats subjected to the CUMS model is crocin (*Crocus sativus* Linn), a compound derived from saffron. Administration of crocin at a dose of 25 mg/kg over a four-week period was shown to increase sucrose consumption and locomotor activity and decrease immobility time in the forced-swim test. In addition, metabolomics tools and network pharmacology were used to identify the major pathways of action of crocin, including biosynthesis of tryptophan, phenylalanine, histidine, glycerolipids, and steroid hormones. These results support the mechanism of action of crocin, which is an alternative treatment for depression [183]. In another study using the CUMS model in rats, the suspension of *Ziziphi spinosae* 100 mg/kg/day for 4 weeks showed an antidepressant effect in the forced-swim test and sucrose consumption. An increase in serum levels of 5-HT and its metabolite 5-hydroxyindoleacetic acid (5HIAA) was also observed. These results could modulate the serotonergic system as a possible mechanism of action [184].

The modulation of the inflammatory process represents an alternative to improve the symptoms of depression. In this sense, the antidepressant effect of the flavonoid quercetin was investigated in a model of LPS-induced depression in rats. Administration of 50 mg/kg quercetin over a 7-day period resulted in an increase in mobility time and grooming time and a decrease in proinflammatory mediators in the brain compared to the LPS-treated group. This suggests that quercetin has antidepressant properties via inhibition of neuroinflammation mediated by modulation of the microglial signaling pathway in the hippocampus and prefrontal cortex of the brain [185].

On the other hand, there are reports on the use of probiotics such as *Lactobacillus* spp., *Bifidobacterium* spp., *Akkermansia* spp., *Clostridium* spp., and *Enterococcus* spp., as a complementary alternative in the pharmacological treatment of depression. Probiotics can modulate the gut–brain axis, inflammation, and the synthesis of various neurotransmitters. In this context, it was reported that 10-week treatment of adult male rats with a mixture of 8 strains of probiotics (a preparation called “Ecologic Barrier”) at a dose of 4.5 g, equivalent to 1.125 × 10^10^ colony-forming units (CFUs), reduced depressive behavior. This treatment reduced depressive-like behavior, improved the immune response, induced changes in the expression of genes related to HPA axis feedback, and increased the expression of genes related to neuroplasticity and neuroprotection, such as BDNF [186]. Similarly, treatment with probiotics in stressed rats restores gut microbiota, an effect that reduces behavioral variables indicative of depression-like behavior and increases levels of NA and 5-HT and inhibits stress hormones such as adrenocorticotropic hormone (ACTH) and corticosterone [187]. In addition, probiotics have shown anxiolytic and antidepressant effects in stress-sensitive rat models that were also subjected to internal deprivation to induce additional stress. In addition, changes in the concentration of DA, 5-HT, and their metabolites were detected in the hippocampus and striatum. These results suggest that treatment with probiotics alters gut flora and brain neurochemistry, an effect that is related to neuroplasticity and emotional state [188].

## 8. Conclusions

Depression is a complex mental disorder that affects millions of people worldwide. Its neurobiology is associated with structural and neurochemical changes in brain regions such as the hippocampus, prefrontal cortex, and amygdala. Preclinical studies of antidepressants and their relationship to neuroplasticity are a very active area of research, reflecting the complexity of depression and contributing to its understanding. These studies suggest that acute antidepressant treatments show immediate effects in behavioral models of depression, such as forced swimming. However, these models have several limitations, as they only allow for the assessment of one characteristic symptom of depression. Further progress in the study of depression and in developing new treatments will be supported by animal models of depression if these were more critically targeted to drug screening vs. studies of underlying neurobiology, clearly stratified to vulnerability and pathogenetic models, focused on well-defined endophenotypes, and validated for each setting while bearing the existing limits to validation in mind. Animal models of depression need not rely merely on behavioral readouts but increasingly incorporate neurobiological measures as the understanding of depression as human brain disorder advances. In addition, the dose, routes of administration, and duration of treatment differ from clinical trials, where chronic treatment is crucial for the activation of intracellular signaling pathways related to neuroplasticity and the establishment of the therapeutic effect. In this sense, the use of animal models represents an approximation of the neuroadaptive mechanisms promoted by conventional antidepressants which increase the bioavailability of various neurotransmitters at the synaptic level. Aspects that are difficult to evaluate in clinical trials for ethical reasons. This review shows the effects of acute and chronic treatment with antidepressants at the preclinical level on neuroplasticity and their relevance for therapeutic efficacy in depression.

## 9. Future Directions

Research on the neurobiology of depression should continue to investigate monoaminergic and non-monoaminergic mechanisms, such as neuroinflammation, changes in the HPA axis, and gut microbiome, and focus on mechanisms related to neuroplasticity. In this context, further preclinical research on the evaluation and efficacy of pharmacological alternatives will allow us to understand the mechanisms involved in the therapeutic effect of depression.

## Figures and Tables

**Table 1:** Classes of antidepressants that are used to treat depression.

Class	Pharmacological Target	Mechanism of Action	Reference
MAOIs			
Selegiline	Monoamine Oxidase Enzyme Type A and B.	Prevents the degradation of 5-HT; increases the availability of 5-HT in the synapses.	[48,49].
Fenelzina			
TCA			
Amitriptyline	5-HT reuptake transporter and NA reuptake transporter.	Inhibits NA and 5-HT reuptake transporters; increases the availability of 5-HT and NA at the synapses; binds to postsynaptic NA, histamine, and acetylcholine receptors.	[45,48].
Imipramine			
Nortriptyline			
SSRIs			
Fluoxetine	5-HT reuptake transporters.	They inhibit the reuptake of 5-HT, which increases the availability of 5-HT in the synapse.	[45,49,58].
Paroxetine			
Escitalopram			
Sertraline			
Specific noradrenergic and serotonergic antidepressants			
Mirtazapine	α2 NA receptors and 5-HT receptors.	α-2 NA receptor antagonists that cause an increased release of 5-HT and NA; they act as antagonists/agonists of 5-HT_2_ and 5-HT_3_ receptors.	[62,68,69,70].
Mianserin			
NaSSAs			
Venlafaxine	5-HT receptors and NA uptake transporters.	Inhibits the reuptake of 5-HT and NA and increases their availability in the synapses.	[70,71].
Duloxetine			
Atypical antidepressants			
Agomelatine	5-HT receptors, NA receptors and melatonin receptors.	Bupropion acts as a DA and NA reuptake inhibitor; vortioxetine acts as an agonist/antagonist of several 5-HT and NA receptors; agomelatine activates melatonin receptors and antagonizes some 5-HT receptors.	[71,72].
Bupropion			
Vortioxetine			
New antidepressants			
Ketamine	Antagonist of the ionotropic glutamate receptor, NMDA 3A. Potentiator of the 5-HT_3A_. Antagonist of the neuronal acetylcholine receptor subunit alpha-7. Inhibitor of nitric oxide synthase brain. Agonist and partial agonist of the dopamine D2 receptor. Agonist of the kappa-type opioid receptor. Antagonist of 5-HT_2_ receptor and 5-HT_1_ receptor.	Ketamine interacts with NMDA receptors, opioid receptors, monoaminergic receptors, muscarinic receptors, and voltage-sensitive Ca ion channels. Unlike other general anesthetic agents, ketamine does not interact with GABA receptors.	[64,65].
Brexenolone	Positive allosteric modulator GABA_A_ receptor.	Brexanolone acts as a barbitu-like positive allosteric modulator of synaptic and extrasynaptic GABA_A_ receptors. In this way, brexanolone may enhance the activity of GABA at these receptors by opening the calcium channels of GABA_A_ receptors more frequently and for longer periods of time. It is also thought that brexanolone triggers this effect on GABA_A_ receptors at a binding site that differs from those of benzodiazepines.	[73,74,75,76].

**Table 2:** Effects of antidepressants drugs on neuroplasticity in animal models.

Drug	Dosage and Experimental Subject	Experimental Model	Effect on Neuroplasticity	Reference
Fluoxetine	10 mg/kg (i.p.). 7 and 14 days of treatment. 7-week-old male C57BL/6 mice.	Tail Suspension Test (TST). Forced-Swim Test (FST). Open-Field Tests (OFT).	Increases Synaptophysin (SYP) expression in hippocampus. Reduction in neuronal deterioration in hippocampal neurons. Increased number of dendritic spines.	[88]
Fluoxetine	15 mg/kg (i.p.). 3 weeks of treatment. CD1 and C57BL/6J mice, homozygous 5-Htt−/− (KO), and 5-Htt+/+ (WT) littermates born from heterozygous (+/−) mutants, bred on a C57Bl/6J background.		Increased expression of BDNF mRNA in the hippocampus. Independent effects of serotonin transporter (5-HTT). Activation of TrkB and CREB proteins in the hippocampus and frontal cortex.	[96]
Ketamine	Ketamine (40 mg/kg/2 h) (i.v.). Ketamine (10 mg/kg/2 h) (i.v.). Adult male Sprague–Dawley rats.	Auditory fear conditioning.	Increased levels of BNDF in amygdala, decreased levels of pERK (extracellular protein kinase regulated by phosphorylation) in the medial prefrontal cortex (mPFC) and hippocampus at doses of 10 mg/kg. 40 mg/kg did not modify BDNF levels, but it did increase pERK in the mPFC and hippocampus, which affects memory-related cell signaling.	[97]
Imipramine	30 mg/kg (v.o.). 3 weeks of treatment. Female WT and *SERT KO* rats.	Elevated plus maze test (EPMT). OFT. Three-chamber social novelty test Puzzle box test. Home-cage activity.	Increased expression of BDNF and its downstream factors such as TrkB and Akt (Protein kinase B) in the infralimbic cortex of *SERT KO* rats.	[98]
Fluoxetine Imipramine	10 mg/kg (i.p.). Male Wistar–Han rats. Two weeks of treatment.	FST. Sucrose Consumption Test (SCT). OFT. Novel Object Recognition (NOR).	Imipramine has a proastrogliogenic effect, promotes differentiation and increases the density of astrocytes in the hippocampus. Fluoxetine induced hypertrophy in astrocytes, imipramine increased the expression of genes related to astrocytic differentiation, such as *STAT3*, *BMP4* and *JMJD3*, in the hippocampus.	[99]
Sertraline	10 mg/kg (i.p.). Sprague–Dawley rats. A single administration.	One-trial inhibitory avoidance task.	Inhibition of long-term potentiation in hippocampal CA1, suggesting a negative effect on neuroplasticity. In addition, the response of NMDA receptors was affected, which inhibits plasticity.	[100]
Sertraline	2.5 and 10 mg/kg administered via a biscuit. Pregnant and non-pregnant female Sprague–Dawley rats.		Increased synaptophysin density in hippocampal regions, specifically in the dentate gyrus (DG) and CA3, in non-pregnant rats. In pregnant rats, neurogenesis in the hippocampus is reduced.	[101]
Venlafaxine	30 mg/kg (i.p.). 14 days. Male C57BL/6J mice and CD1 mice.	CUMS Chronic social defeat stress (CSDS). TST. FST. SPT. Social interaction test.	It prevents the reduction in the expression of mTORC1 signaling markers, such as p-mTORC1, p-4E-BP-1 and p-p70S6K, in the hippocampus of mice subjected to CSDS. In addition, it increased the expression of these markers in control mice, suggesting that venlafaxine activates the mTORC1 signaling cascade. Increased BDNF levels in the hippocampus.	[102]
Escitalopram	5 and 10 mg/kg (v.o.). 10 days of treatment. Male Wistar rats.	CUMS. EPMT. SPT.	Reduction in MDA levels in the hippocampus and frontal cortex. Increased levels of Glutathione reductase (GSH) and GSH/Glutathione disulfide (GSSG) in the hippocampus. Decreased caspase-3 activity and regulates BDNF and MeCP2 levels	[103]
Mirtazapine	10 mg/kg (i.p.). 21 days of treatment. Pregnant Sprague–Dawley females.	Social interaction test. Novel object recognition test.	Increased BNDF expression in the hippocampus and frontal cortex.	[104]
